# Brain Glycogen—Its Metabolic Role in Neuronal Health and Neurological Disorders—An Extensive Narrative Review

**DOI:** 10.3390/metabo15020128

**Published:** 2025-02-13

**Authors:** Ana Isabel Beltran-Velasco

**Affiliations:** NBC Group, School of Life and Nature Sciences, Nebrija University, 28248 Madrid, Spain; abeltranv@nebrija.es

**Keywords:** brain glycogen, neuronal health, neurodegenerative diseases, epilepsy, glucose metabolism, glycogen metabolism, neuroglia, enzyme modulation, therapeutic strategies

## Abstract

**Background:** Brain glycogen is imperative for neuronal health, as it supports energy demands and metabolic processes. This review examines the pathways involved in glycogen storage and utilization in the central nervous system, emphasizing their role in both physiology and pathology. It explores how alterations in glycogen metabolism contribute to neurological disorders, including neurodegenerative diseases, epilepsy, and metabolic conditions while highlighting the bidirectional interaction between neurons and glia in maintaining brain homeostasis. **Methods:** A comprehensive search of articles published between 2015 and 2025 was conducted using the following databases: ScienceDirect, Scopus, Wiley, Web of Science, Medline, and PubMed. The selection of relevant studies was based on their focus on brain glycogen metabolism and its role in neurological conditions, with studies that did not meet the inclusion criteria being excluded. **Results:** The metabolic processes of brain glycogen are subject to rigorous regulation by astrocyte–neuron interactions, thereby ensuring metabolic homeostasis and energy availability. The dysregulation of glycogen storage and mobilization has been implicated in the development of synaptic dysfunction, excitotoxicity, and neurodegeneration in a variety of disorders. For instance, aberrant glycogen accumulation in diseases such as Lafora disease has been associated with severe neurodegeneration, while impaired glycogen mobilization has been shown to exacerbate energy deficits in Alzheimer’s and epilepsy. **Conclusions:** Targeting brain glycogen metabolism represents a promising approach for therapeutic intervention in neurological disorders. However, the translation of these strategies to human models remains challenging, particularly with regard to the long-term safety and specificity of glycogen-targeted therapies.

## 1. Introduction

Energy metabolism is critical to the functioning of the central nervous system (CNS). Despite representing only 2% of body weight, the CNS consumes approximately 20% of the body’s total energy [1]. Within this intricate metabolic system, brain glycogen has emerged as a key player. Historically, its relevance was underestimated due to low relative concentrations compared to other tissues, such as the liver and skeletal muscle [2,3].

Glycogen in the brain is predominantly found in astrocytes, where it functions as a strategic energy reserve that can be rapidly mobilized to meet the energy demands of neurons [4]. Beyond its classical role as a glucose store, recent research has revealed that brain glycogen is actively involved in essential physiological processes such as synaptic plasticity, learning, and memory, and plays a critical role in brain homeostasis under conditions of metabolic stress, hypoglycemia, or injury [5,6].

From a physiological perspective, a comprehensive understanding of glycogen metabolism in the brain is imperative, as it bears significant pathological implications [7]. Alterations in this system have been associated with a variety of neurological disorders, including epilepsy, neurodegenerative diseases, and congenital metabolic disorders, underscoring its potential as a therapeutic target [8].

### 1.1. Role of Glycogen Metabolism in the Brain

Glycogen, long recognized as the primary energy reserve in vital tissues such as the liver and muscles, has recently emerged as a pivotal player in the CNS. In the CNS, glycogen is predominantly found in astrocytes, a type of glial cell that is critical for providing metabolic support to neurons [9,10]. This polysaccharide is stored in these cells, where it is rapidly mobilized in response to specific energy demands, including periods of hypoglycemia, intense neuronal activity, or metabolic stress [11].

Recent findings have demonstrated the presence of low levels of glycogen in neurons, oligodendrocytes, and microglia. However, the contributions of these cells to the overall brain energy metabolism remain to be fully elucidated, with their roles likely being secondary to the astrocytic glycogen pool. These observations indicate that glycogen metabolism within the brain is a more intricate and dynamic process than previously recognized. It is postulated that distinct cell populations may contribute to this process in varying degrees, contingent on specific physiological or pathological conditions [12]. Astrocytes, in contrast to neurons, possess both the capacity to accumulate glycogen and convert it into lactate, a pivotal metabolite that neurons require during periods of elevated metabolic demand. This lactate, which is produced through the process of glycogenolysis, is subsequently transferred to neurons via specific transporters, underscoring the metabolic interdependence between these two cell populations [13,14,15].

Beyond energy provision, glycogen plays critical roles in synaptic plasticity, regulation of the sleep–wake cycle, and response to injury or metabolic stress. Recent studies have shown that glycogen-derived lactate is essential for memory consolidation in the hippocampus [16,17]. This metabolite serves as an alternative energy substrate and regulates intracellular signaling pathways involved in synaptic connection stabilization. Glycogen metabolism fluctuates throughout the sleep–wake cycle [18]. During sleep, glycogen levels are restored, while prolonged wakefulness leads to depletion [19]. This pattern indicates that glycogen is crucial for metabolic recovery and neuronal homeostasis, with implications for sleep physiology and sleep deprivation-related disorders [20,21].

A crucial function of brain glycogen is providing immediate energy during ischemic events or oxidative stress. In the absence of oxygen, glycogen stored in astrocytes undergoes anaerobic glycolysis, generating a transient energy supply essential for cell viability and preventing neuronal damage [22]. This response is critical in pathological contexts like stroke or severe hypoglycemia, where glycogen stores serve as a metabolic buffer, protecting neurons from cell death [23].

Glycogen metabolism is also intrinsically linked to the metabolic interaction between astrocytes and neurons [24]. This concept, termed the astrocyte–neuron metabolic unit, emphasizes astrocytes’ role in regulating energy availability through glycogen storage and mobilization, as well as their role in modulating neuronal activity. These interactions are crucial for maintaining brain function and may contribute to neurological pathologies when altered [25,26].

Moreover, recent research has revealed that its function extends far beyond this historical perspective. Beyond its role in supplying energy during periods of stress, glycogen exhibits a direct involvement in critical neurological processes, underscoring its significance in both cerebral health and pathologies [27]. This knowledge has initiated several research advances, primarily focused on investigating the mechanistic underpinnings through which alterations in glycogen metabolism contribute to the onset of neurological diseases and on identifying potential therapeutic interventions that are predicated on its modulation [4,28,29].

### 1.2. Physiological and Pathological Implications

The study of brain glycogen has garnered increasing interest in recent decades, given its foundational role in CNS energy homeostasis and its involvement in various pathological conditions. Historically, brain glycogen concentrations were considered low and functionally insignificant compared to those of other organs such as the liver and muscle. However, recent research has shown that this molecule has essential functions in brain tissue, particularly in astrocyte–neuron interactions [30,31].

In the physiological context, glycogen serves as an energy reserve that can be rapidly mobilized to meet high neuronal demands, such as during learning, memory, or stress responses [32,33]. These functions make it critical for synaptic plasticity and the maintenance of neuronal activity, especially when the glucose supply may be inadequate [34,35].

The regulation of brain glycogen is inextricably linked to the brain’s capacity to adapt to metabolic fluctuations [36,37]. During sleep, glycogen stores are restored after being partially depleted during periods of prolonged wakefulness [38,39]. This underscores the critical role of glycogen as an energy buffer, facilitating brain function recovery over extended periods. The interplay between glycogen metabolism and the sleep–wake cycle has prompted significant research on its impact on cognitive processes like memory consolidation and its dysfunction in sleep disorders and age-related cognitive decline [40,41].

Glycogen metabolism plays a role in several neurological diseases. Alterations in glycogen synthesis, storage, or degradation are associated with disorders like Lafora disease, a progressive epilepsy marked by abnormal glycogen accumulation in the brain [42]. Additionally, changes in glycogen metabolism have been identified in neurodegenerative diseases, including Alzheimer’s disease and amyotrophic lateral sclerosis (ALS), where its dysfunction contributes to metabolic imbalance and neuronal damage [43,44].

The response of brain glycogen to ischemic or hypoxic events has also been studied. In extreme stress situations, glycogen provides energy through anaerobic pathways, maintaining cell viability and reducing neuronal damage [45]. However, its rapid mobilization can lead to an accumulation of by-products like lactate, which, in excess, may contribute to tissue acidosis and exacerbate brain damage [46]. This balance highlights the need to explore the mechanisms regulating glycogen metabolism in pathological contexts and its potential as a therapeutic target [47,48].

The study of brain glycogen has the potential to facilitate advancements in our understanding of the fundamental mechanisms underlying neurophysiology. It also facilitates novel perspectives for addressing neurological diseases from a metabolic perspective.

This review discusses the critical role of brain glycogen in neuronal health and its involvement in neurological disorders. It explores glycogen’s contributions to normal brain function and its impact on diseases such as neurodegenerative diseases, epilepsy, and sleep disorders. The main objective is to examine the molecular mechanisms regulating brain glycogen metabolism and explore therapeutic strategies aimed at restoring homeostasis in these diseases. By emphasizing the significance of brain glycogen as a crucial component of neuronal health and its potential as a therapeutic target, this article provides a relevant perspective in neuroscience.

## 2. Physiological Functions of Glycogen in the Brain

Brain glycogen is also imperative for sustaining optimal physiological processes, thereby ensuring brain health. Recent research findings have elucidated the pivotal role of glycogen in diverse brain functions, ranging from energy regulation to synaptic plasticity and memory [4,49].

### 2.1. Energy Source During Hypoglycemia

Brain glycogen has been identified as a key source of energy in the brain, especially during periods of hypoglycemia [50]. During episodes of low blood glucose, the brain relies on its glycogen stores to maintain a constant supply of energy to neurons, as these cells have a very high energy requirement and their function is seriously compromised in the absence of glucose or an adequate alternative source of energy [51].

When glucose levels decline, astrocytes, which store substantial amounts of glycogen, mobilize this glycogen through a process known as glycogenolysis. This process releases lactate and glucose-1-phosphate, which are then transported to neurons [52]. In the neurons, lactate is converted into pyruvate, which is subsequently used in the respiratory chain to produce ATP, thereby ensuring the continuity of neuronal activity. This process is critical for the maintenance of basic neuronal functions, as well as for ensuring synaptic stability and neuroplasticity during periods of energy shortage [53].

Recent studies have demonstrated that the communication between astrocytes and neurons during hypoglycemia is facilitated by intracellular signaling mechanisms that regulate the activation of enzymes responsible for glycogenolysis, such as phosphorylase [54]. This enables a swift response to fluctuations in glucose availability and efficient adaptation of brain metabolism. The plasticity of these compensatory mechanisms is regarded as a pivotal factor in preventing neuronal damage in conditions of chronic or acute hypoglycemia [55].

### 2.2. Role in Synaptic Plasticity and Memory

Synaptic plasticity, which is the term given to the ability of synapses to strengthen or weaken in response to neuronal activity, is a fundamental process in memory consolidation and learning. Glycogen serves two primary functions, as an energy source and in the modulation of synaptic activity and plasticity through metabolic and signaling mechanisms [56].

The impact of astrocytes on synaptic plasticity has been demonstrated by previous studies, which have shown that the ability of these cells to store and release glycogen directly impacts synaptic plasticity [57]. During periods of intense synaptic activity such as those observed during learning processes, astrocytes release lactate, which is subsequently taken up by neurons and utilized for ATP synthesis [58]. This ATP, in turn, plays a critical role in the phosphorylation of key proteins in synapses, thereby promoting long-term potentiation (LTP), a phenomenon associated with the strengthening of neuronal connections and the formation of memories [4,59].

Research in animal models has demonstrated that manipulating glycogen metabolism in astrocytes can modify the capacity of neurons to establish new synaptic connections [60]. Inhibition of glycogenolysis in astrocytes has exhibited a detrimental effect on synaptic plasticity in brain regions that are critical for memory, such as the hippocampus, thereby underscoring the significance of this process in the consolidation of long-term memory [61,62]. These findings suggest that the regulation of brain glycogen is not only essential for maintaining neuronal activity but also for learning and memory.

### 2.3. Regulation of the Sleep–Wake Cycle

The sleep–wake cycle is another brain process in which glycogen plays a fundamental role. During sleep, especially in the deep and REM sleep phases, the brain experiences a decrease in overall metabolic activity. However, some areas of the brain, such as the hippocampus, remain active and require a constant supply of energy, causing glycogen to become a critical source of energy during sleep [6,63]. The neuronal activity in these regions, which are implicated in the consolidation of memories, is contingent on the glycogen stored in astrocytes, which is essential for maintaining efficient synaptic activity [64].

During sleep, the brain exhibits a significantly faster rate of glycogen consumption compared to periods of wakefulness. This observation suggests that glycogen plays a pivotal role in the process of cleaning neuronal connections and in the consolidation of memories acquired during the day [65]. This dynamic is facilitated by the activity of glia, which helps regulate the release of glycogen and its subsequent conversion into lactate. Lactate serves as a source of energy and as a modulator of neuronal activity during sleep [66].

Conversely, an association has been demonstrated between alterations in brain glycogen regulation and the development of sleep disorders, including insomnia, in patients afflicted with neurological diseases. For instance, in animal models of epilepsy and Alzheimer’s disease, a decline in glycogen levels has been observed in critical regions of the brain during sleep [24,67]. This observation suggests the potential involvement of altered glycogen metabolism in the pathophysiology of sleep disturbances in these neurological conditions.

### 2.4. Glycogen in Energy Compensation After Brain Injury

Following brain injury, energy homeostasis is severely disrupted, requiring compensatory mechanisms to restore neuronal function. Glycogen, primarily stored in astrocytes, plays a crucial role in this recovery process by serving as an emergency energy reservoir. In response to ischemic or traumatic brain injury, glycogen metabolism undergoes rapid changes, including increased breakdown via glycogenolysis to provide lactate and glucose as alternative energy substrates for neurons [68]. Studies have demonstrated that astrocytic glycogen degradation is a feature of hypoxic conditions, supplying neurons with metabolic fuel to sustain their function and enhance survival [69]. Furthermore, glycogen mobilization contributes to neuroprotection by maintaining synaptic activity and preventing excitotoxic damage, which is a common consequence of energy depletion in injured brain regions. Given these findings, strategies targeting glycogen metabolism may hold therapeutic potential for enhancing post-injury recovery and reducing neuronal loss [70,71].

## 3. Materials and Methods

The present article focuses on the mechanisms by which brain glycogen modulates neuronal function, synaptic plasticity, and response to metabolic stress events, and how alterations in this metabolism may contribute to neuronal and glial dysfunction in different diseases. The most relevant advances in glycogen metabolism are analyzed, highlighting its role in the interaction between astrocytes and neurons, its function as an energy source, and its impact on neuroinflammation and oxidative stress.

In order to ensure the relevance and quality of the reviewed studies, including those addressing brain glycogen in the context of its influence on neuronal function and brain health, inclusion and exclusion criteria were established. Studies that did not explicitly address glycogen metabolism or were not directly related to neurological disorders, including non-exclusively neurodegenerative conditions, were excluded. The search was conducted in recognized scientific databases such as ScienceDirect, Scopus, Wiley, Web of Science, Medline, and PubMed, limited to studies published between 2015 and 2025, with emphasis on research exploring innovations related to glycogen metabolism in brain health. The methodology previously employed in other extensive literature reviews was adhered to [72,73].

To optimize the search and ensure comprehensive coverage, Boolean operators, and combinations of key terms were used such as (cerebral glycogen OR brain glycogen) AND (neurodegeneration OR neurological disorders OR neuroinflammation OR epilepsy OR Alzheimer’s OR Parkinson’s OR stress response) AND (astrogliosis OR glia OR astrocytes) AND (metabolism OR biomarkers OR therapy) AND (synaptic plasticity OR memory consolidation OR neuroprotection).

## 4. Overview of Glycogen Metabolism in the Brain

In recent years, there has been a marked increase in the focus on brain glycogen due to its critical role in neuronal function and various CNS pathologies. Historically, the association of glycogen primarily lay with its energetic function in muscle and liver. However, recent research has revealed the multifaceted and more complex roles of glycogen in the brain. Recent research has significantly advanced our understanding of its localization, regulation, and dynamics, providing new insights into its relevance in neuroscience, especially with regard to its interaction with glial cells and neurons [74,75].

### 4.1. Localization and Dynamics of Glycogen in CNS

Brain glycogen is found in various cell types within the CNS, including astrocytes, neurons, microglia, and oligodendrocytes, although its concentration varies among these cells. Astrocytes, due to their high storage capacity, play a pivotal role in maintaining brain energy homeostasis. However, recent studies have highlighted that neurons and other glial cells also store glycogen, albeit in smaller amounts, and are involved in local energy metabolism, especially under conditions of metabolic stress or high neuronal activity. The dynamic regulation of glycogen stores in these cells is crucial for sustaining brain function during fluctuations in energy demand [76].

The distribution of glycogen within the CNS exhibits heterogeneity, with higher concentrations observed in metabolically active regions such as the hippocampus, which plays a role in learning and memory processes. The synthesis and breakdown of glycogen are subject to rigorous regulation by enzymes such as glycogen synthase and glycogen phosphorylase, ensuring the efficient mobilization of glycogen stores in accordance with the energy demands of the brain [77].

Various animal models have been instrumental in enhancing our comprehension of glycogen metabolism within the brain. Rodent models, most notably, mice and rats, have been extensively utilized to investigate the manner in which glycogen is utilized during periods of elevated metabolic demand, such as learning, memory consolidation, or following brain injury. These models have facilitated the acquisition of knowledge regarding the mechanisms that govern glycogen storage and mobilization, including the role of astrocytes in supplying lactate to neurons during periods of low glucose availability. Furthermore, genetic modifications that alter glycogen synthesis or degradation in specific cell types have enabled researchers to examine the functional consequences of glycogen metabolism dysregulation in neurological conditions.

For example, transgenic mouse models with impaired glycogen metabolism in astrocytes or neurons have revealed how such alterations contribute to cognitive dysfunction, neuronal damage, and neurodegenerative diseases like Alzheimer’s disease and amyotrophic lateral sclerosis (ALS). Furthermore, models of brain injury, such as stroke, have demonstrated the critical role of glycogen as a neuronal energy reserve, protecting against ischemic damage. These findings underscore the metabolic interdependence between astrocytes and neurons, emphasizing the importance of glycogen in maintaining brain function under both physiological and pathological conditions [78,79].

Brain glycogen is predominantly found in astrocytes, although it is also present to a lesser extent in neurons, microglia, and oligodendrocytes [52]. Astrocytes, due to their high capacity to store glycogen, are primarily responsible for regulating glucose levels in the brain. Glycogen in these astrocytes fulfills two distinct functions. Firstly, it serves as a rapid source of energy during periods of high metabolic demand. Secondly, it participates in intracellular signaling and modulation of neuronal function [53]. Astrocytes fulfill an essential role in supplying neurons with lactate through the so-called astroglial–neuronal cycle, a pivotal mechanism that enables neurons to sustain their metabolic activity when glucose levels are limited or under duress [12].

Glycogen is heterogeneously distributed within the brain, predominantly in cortical and subcortical regions, and its distribution fluctuates in accordance with the functional activity of specific brain areas. In the most metabolically active regions, such as the hippocampus, a higher concentration of glycogen has been observed, suggesting a potential involvement in memory and learning processes [54]. Glycogen dynamics are subject to a delicate balance between its synthesis and degradation, regulated by a series of key enzymes that modulate its availability according to the brain’s energy needs [55].

### 4.2. Structure and Biochemical Properties of Glycogen

Glycogen, a highly branched polysaccharide, is composed of glucose molecules linked by α-1,4-glycosidic bonds, with branch points formed by α-1,6-glycosidic bonds approximately every 8–12 glucose residues. This structural configuration enables glycogen to function as a compact and highly accessible energy reserve. The branching of glycogen increases its solubility and provides multiple sites for enzymatic action, facilitating rapid mobilization when energy is required. These properties render glycogen particularly well-suited for its role as a rapidly mobilized energy source, which is essential for maintaining cellular homeostasis, particularly in tissues with high energy demands, such as the brain [80,81].

At the molecular level, glycogen exists in the form of granules that are associated with a complex of enzymes and regulatory proteins. This glycogen–protein complex plays a critical role in the control of glycogen metabolism, with enzymes such as glycogen synthase and glycogen phosphorylase dictating its synthesis and breakdown, respectively. The activity of these enzymes is further regulated by proteins such as protein phosphatase-1 and AMP-activated protein kinase (AMPK), which fine-tune the activity of these enzymes in response to the cellular energy status. The phosphorylation state of these enzymes plays a pivotal role in balancing glycogen storage and degradation, thereby ensuring that the mobilization of energy occurs precisely when it is required [82,83].

In the context of astrocytes, the strategic positioning of these granules in close proximity to mitochondria and synaptic terminals is of paramount importance, ensuring efficient energy transfer to neurons. This spatial organization assumes particular significance during periods of high neuronal activity when astrocytes play a crucial role in maintaining neuronal energy balance. The coupling of glycogen metabolism to mitochondrial function facilitates a rapid supply of energy through the release of lactate, a byproduct of glycogen degradation that is shuttled to neurons via the astrocyte–neuron lactate shuttle. This mechanism is essential for sustaining synaptic function and neurotransmitter recycling, particularly under conditions of metabolic stress, such as during intense neuronal firing or periods of hypoglycemia [84,85].

Beyond its role in energy storage, glycogen granules exhibit biochemical properties that extend to signaling pathways regulating processes beyond metabolism. These pathways include protein synthesis, cell growth, and autophagy. The interaction between glycogen and signaling molecules such as AMP-activated protein kinase (AMPK) and the mechanistic target of rapamycin (mTOR) can influence these processes [86].

The solubility and enzymatic accessibility of glycogen are therefore critical for its role as an energy buffer in the central nervous system, which enables it to respond dynamically to fluctuating energy demands, particularly in highly active regions of the brain. The functional dynamics of glycogen metabolism in the brain are intricate, involving the coordination of multiple metabolic pathways that ensure neurons remain energetically supported while astrocytes maintain their pivotal role in energy regulation [6,87].

The properties of glycogen are such that it plays a pivotal role in neuronal function, particularly in the context of diseases that are characterized by energy deficits. Such diseases include epilepsy, Alzheimer’s disease, and other neurodegenerative conditions. In these disorders, impairments in glycogen metabolism or availability can lead to neuronal dysfunction, contributing to cognitive decline, synaptic failure, and exacerbation of neuroinflammation [88]. A more profound understanding of the structural and biochemical properties of glycogen could identify new therapeutic avenues for restoring normal brain energy metabolism in these conditions, which could have significant implications for the treatment of neurological disorders [89].

#### Enzyme Regulation—Glycogen Synthase, Phosphorylase, and Their Modulators

The enzymatic regulation of brain glycogen is critical to maintaining an optimal balance between glycogen synthesis and degradation. Glycogen synthase and phosphorylase are the two primary enzymes responsible for regulating these processes, and their activity is modulated by a series of intracellular and extracellular factors [90].

Glycogen synthase, the enzyme responsible for synthesizing glycogen from glucose-6-phosphate, plays a pivotal role in regulating brain glycogen levels. Its activity is subject to modulation by phosphorylation at various sites, which adjusts its activity in accordance with the metabolic conditions of the brain [91]. In conditions of high glucose availability, glycogen synthase is activated, promoting the synthesis of glycogen for storage in astrocytes. Conversely, in situations of energy stress or during periods of intense neuronal activity, glycogen synthase is inhibited, facilitating the release of stored glycogen [92].

Phosphorylase, conversely, is the enzyme responsible for the breakdown of glycogen to glucose-1-phosphate, which can be used in the generation of ATP or converted to lactate for use by neurons. Its activation is also regulated by phosphorylation, and its action is particularly relevant during periods of high metabolic demand, such as in the learning process or during episodes of hypoxia [93]. Furthermore, phosphorylase is modulated by a number of factors, such as AMP-activated protein kinase (AMPK), which responds to reductions in cellular energy, and intracellular calcium, which plays a crucial role in the activation of phosphorylase in contexts of elevated neuronal activity [94].

Beyond the enzymes, a multitude of modulators influence the regulatory processes of glycogen metabolism. Among these modulators are hormones such as insulin and glucagon, as well as protein kinases. These kinases respond to diverse metabolic and neuronal signals within the body. This intricate regulatory network ensures the brain’s glycogen is consistently available to meet the metabolic demands of neurons and other CNS cell types [95].

### 4.3. Methods to Study Brain Glycogen

Recent advancements in the domain of neuroscience have precipitated a significant advancement in the study of brain glycogen metabolism. The introduction of innovative techniques has enabled researchers to approach glycogen metabolism from multiple perspectives, thereby facilitating the acquisition of more comprehensive and precise data on its dynamics, location, and role in brain function [96].

Imaging techniques have emerged as particularly powerful tools in the realm of brain glycogen research. The utilization of methods such as proton magnetic resonance imaging (MRS) and positron emission tomography (PET) enables the measurement of brain glycogen concentrations in vivo, thereby obviating the necessity for invasive procedures such as biopsies. These methodologies, when complemented by magnetic resonance spectroscopy, facilitate the mapping of glycogen distribution across different brain regions, thereby providing critical insights into its localization and variations under both physiological and pathological conditions [97,98].

Conversely, metabolomics analysis has transformed the study of glycogen-derived metabolites such as lactate and glucose [99]. Techniques like liquid chromatography coupled with mass spectrometry (LC-MS) enable the precise and real-time measurement of these metabolites, facilitating the understanding of how glycogen metabolism affects neuronal bioenergetics and synaptic plasticity [100].

Animal models continue to be an essential tool for studying the effects of glycogen metabolism in the brain. Through genetic manipulation, mouse models have been created with alterations in key enzymes of glycogen metabolism, allowing researchers to examine the impact of these changes on brain function, memory, and behavior, as well as the development of diseases [101,102].

Notwithstanding the notable advancements in the field, there persist significant limitations in the study of brain glycogen. A substantial challenge pertains to the difficulty in accurately quantifying glycogen levels in the brain in vivo. Although imaging and metabolomics techniques have been advanced, they continue to exhibit limitations in terms of spatial resolution and sensitivity in detecting subtle changes in glycogen concentration.

**Figure 1:**
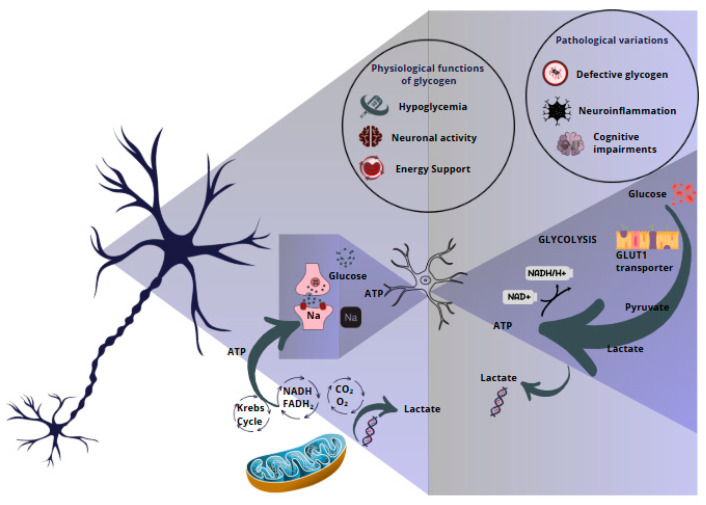
The role of brain glycogen in astroglia-neuronal interaction. Physiological functions and pathological alterations. The metabolism of brain glycogen and its relevance to the interaction between astrocytes and neurons. Glycogen stored in astrocytes is degraded to lactate, which is subsequently transferred to neurons as an energy source for functions such as synaptic plasticity and maintenance of homeostasis during hypoglycemia. Under pathological conditions, alterations in glycogen metabolism, such as the accumulation of defective inclusions, contribute to neurodegeneration and energy dysfunctions.

Therefore, research on the interaction between astrocytes and neurons in glycogen metabolism remains in its nascent stages. Despite the strides made in elucidating this process, numerous underlying mechanisms remain to be fully elucidated, underscoring a substantial knowledge gap (Figure 1).

## 5. Alterations of Glycogen Metabolism in Neurological Disorders

Brain glycogen metabolism is critical for CNS health and function. However, in several neurological pathologies, alterations in glycogen regulation and storage may contribute to the development and progression of these pathologies. A correlation between dysfunction in proper glycogen utilization and a range of neurological disorders, including epileptic disorders, neurodegenerative diseases, and congenital metabolic disorders, has been established.

### 5.1. Epilepsy

Epilepsy, a prevalent neurological disorder, is characterized by the occurrence of recurrent seizures due to aberrant neuronal excitability. In this context, brain glycogen assumes a pivotal role, serving as a crucial energy source during episodes of elevated metabolic demand, such as seizures [103,104]. Astrocytes, which store glycogen, undergo a transition in metabolism, releasing lactate during periods of intense neuronal activity, a process that is imperative for sustaining neuronal function [105]. However, recent research suggests that abnormal glycogen accumulation in astrocytes may contribute to the excessive neuronal excitability observed in epilepsy, especially in certain types of refractory epilepsy [106,107].

The alteration of glycogen metabolism in epilepsy may be due to a dysfunction in the enzymatic regulation of the main enzymes involved in the synthesis and degradation of glycogen, such as glycogen synthase and phosphorylase [108]. This dysfunction has the potential to modify the capacity of astrocytes to effectively release glycogen, thereby affecting the energy homeostasis of neurons and increasing their susceptibility to seizures [29,109]. Recent studies in animal models have demonstrated that modifying these enzymatic mechanisms by manipulating the activity of these enzymes could reduce the frequency and intensity of seizures, suggesting a potentially effective therapeutic approach [110,111].

In this line, the ketogenic diet, a dietary modification that induces a state of ketosis and affects energy metabolism in the brain, has demonstrated beneficial effects in the treatment of epilepsy, in part due to its effects on brain metabolism [112]. Research in this area continues to be critical, with a focus on understanding how manipulation of glycogen availability in the brain can influence neuronal excitability and seizure control [113,114,115,116].

### 5.2. Neurodegenerative Diseases

Neurodegenerative diseases are characterized by the progressive loss of neuronal function and a hallmark of these pathologies is the disruption of brain energy metabolism [117]. Specifically, research has identified brain glycogen and its metabolism in astrocytes as a primary focus of study, as impaired glycogen utilization may contribute to neurodegeneration.

In Alzheimer’s disease, astrocytes play a crucial role in regulating the metabolic environment of the brain. However, it has been observed that in the early stages of the disease, glycogen metabolism in astrocytes is altered [118]. The accumulation of tau protein and beta-amyloid plaques in the brains of Alzheimer’s patients has been documented to impair the ability of astrocytes to store and release glycogen, contributing to energy dysfunction that affects both neurons and glial cells [119]. The alteration of glycogen metabolism in this disease compromises neuronal energy homeostasis and favors the development of a neuroinflammatory environment that exacerbates neurodegeneration [120,121].

In Parkinson’s disease, impaired glycogen metabolism has been associated with basal ganglia damage, where energy balance is imperative for motor control [122]. Modifications in glycogen within astrocytes in this brain region have been demonstrated to impede the capacity of dopaminergic neurons to sustain optimal function, thereby contributing to the manifestation of the disease’s motor symptoms [123,124]. Therapeutic approaches aimed at restoring glycogen regulation have emerged as a promising avenue for addressing both the motor symptoms and cognitive deficits associated with Parkinson’s disease.

In Huntington’s disease, a genetic disorder characterized by progressive motor dysfunction, cognitive decline, and psychiatric symptoms, abnormalities in glycogen metabolism have also been implicated. Studies suggest that mutant huntingtin protein disrupts astrocytic metabolic functions, including glycogen storage and breakdown [125,126,127]. This metabolic imbalance exacerbates neuronal energy deficits, contributing to neurodegeneration in the striatum and cortex, the regions most affected by the disease. Furthermore, altered glycogen dynamics have been associated with increased oxidative stress and mitochondrial dysfunction, which further exacerbates neuronal death [128].

As research in this field continues to advance, studies exploring the modulation of enzymes involved in glycogen metabolism and the enhancement of mitochondrial function in glial cells may unveil novel therapeutic strategies for the treatment of these diseases [119]. Furthermore, an increasing body of evidence highlights the pivotal role of glycogen metabolism in the context of neuroinflammation, a well-established contributor to neurodegeneration [129,130,131]. This underscores the imperative to target these metabolic processes in the development of effective therapeutic interventions.

### 5.3. Congenital Metabolic Disorders

Congenital glycogen-related metabolic disorders are a rare but serious group of diseases that affect the synthesis and degradation of glycogen in various tissues, including the brain. In this line, Lafora disease is an inherited disorder characterized by the accumulation of abnormal glycogen in cells, causing severe neurological symptoms, such as seizures and dementia [132]. This condition arises from genetic mutations that impair the processes of glycogen formation and degradation. A hallmark feature of Lafora disease is the presence of Lafora bodies, which are structurally defective glycogen inclusions that accumulate in various tissues, including the brain and heart [133,134].

The defective glycogen metabolism in these disorders precipitates neuronal and glial dysfunction, thereby disrupting the energy balance of the brain and contributing to the progressive neuronal degeneration observed in these patients [135]. The therapeutic landscape for Lafora disease and other congenital glycogen-related metabolic disorders is limited at present, with available treatment options including dietary interventions and pharmacological treatments that aim to manage symptoms [136,137]. However, research focusing on the molecular correction of glycogen metabolism holds considerable promise as a therapeutic avenue for these conditions.

Recent advancements in the comprehension of the molecular mechanisms underlying glycogen-related metabolic disorders have given rise to novel therapeutic opportunities. In addition to addressing the issue of abnormal glycogen accumulation, emerging studies are exploring the broader implications of modulating metabolic imbalances in neuronal and glial cells [138,139]. These endeavors aim not to mitigate neurodegeneration but rather to restore cellular function and intercellular communication, which are disrupted by metabolic dysfunction [132].

**Figure 2:**
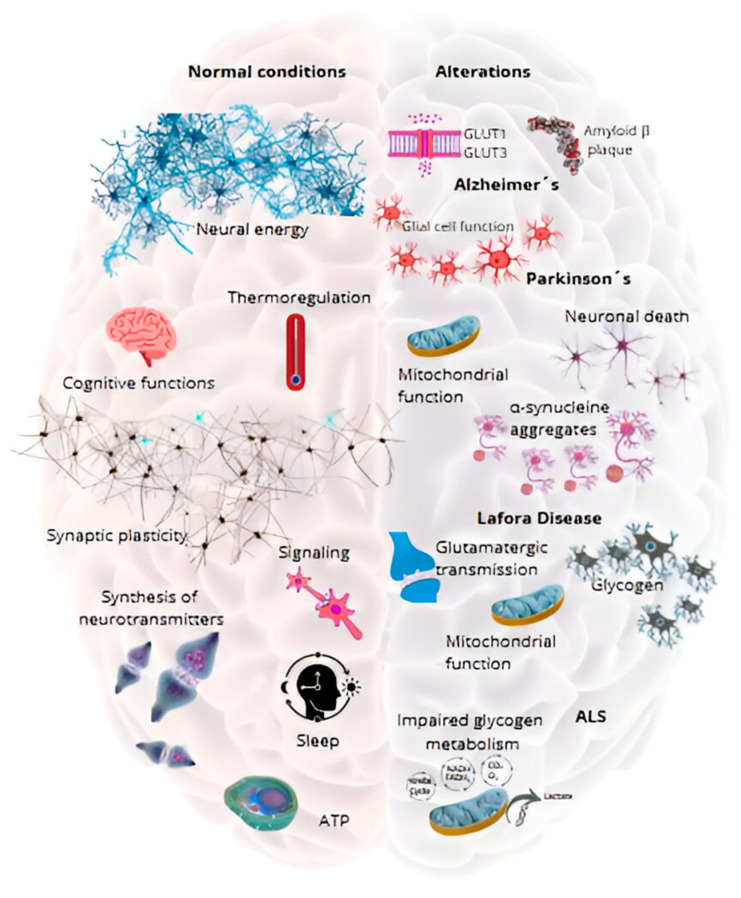
Functions of brain glycogen and its alteration in neurological diseases. Physiological functions of glycogen in the brain (its role in energy supply, synaptic plasticity, and regulation of the sleep–wake cycle), and metabolic alterations associated with neurological diseases (e.g., Alzheimer’s, Parkinson’s, Lafora).

In this regard, the modulation of pathways associated with oxidative stress and inflammation, which are exacerbated by glycogen metabolism abnormalities, has the potential to offer complementary benefits. Innovative approaches, such as the development of small molecules to regulate glycogen synthase activity and the use of advanced gene editing technologies, are showing promising results in preclinical models [140,141]. Consequently, these findings underscore the potential for a multifaceted strategy to treat congenital glycogen-related metabolic disorders, integrating direct correction of alterations in glycogen metabolism with broader neuroprotective interventions (Figure 2).

## 6. Glycogen and Neuroglial Communication—Establishing a Metabolic Bridge

Brain glycogen is an essential component in the interaction between neurons and glial cells, playing a fundamental role in the regulation of brain metabolism and energy homeostasis. Astrocytes, as the main cells responsible for storing and regulating glycogen in the brain, play a crucial role in supplying energy to neurons [142,143]. This dynamic interplay between neurons and glia, facilitated by glycogen and its metabolites, is critical for sustaining normal brain function and for acclimating to variations in metabolic conditions or periods of stress. Moreover, this interaction exerts a direct influence on the modulation of pathological processes, including neuroinflammation and dysfunction in various neurological diseases [5,144].

A pivotal mechanism underlying the communication between neurons and astrocytes entails the exchange of lactate, a by-product of glycogen metabolism within astrocytes [138]. These cells store glycogen and undergo glycogenolysis to release lactate and glucose-1-phosphate, two metabolites that neurons utilize as energy sources [145]. In particular, lactate has been identified as a crucial substrate for neurons, given their limited capacity to store glycogen and their reliance on lactate from glial cells to maintain their metabolic activity [146,147].

Recent findings have demonstrated that lactate transfer from astrocytes to neurons is essential for brain function, particularly during periods of high neuronal activity. During learning and memory processes, astrocytes release lactate, which is subsequently taken up by neurons and utilized in the mitochondria to generate ATP [148]. This process contributes to memory consolidation and synaptic plasticity. Dysfunction in this metabolic exchange between glia and neurons can contribute to various neurological pathologies, such as epilepsy and neurodegenerative diseases, where the ability of astrocytes to provide lactate to neurons is compromised [149,150].

Research in this area indicates that alterations in glycogen metabolism and the ability of astrocytes to release lactate may be associated with a decrease in synaptic activity in brain areas critical for learning and memory, such as the hippocampus [151,152]. These findings suggest that impaired neuroglial communication could be a key factor in the development of cognitive deficits in several diseases.

### 6.1. The Function of Glia in the Modulation of Neuroinflammation and Brain Metabolism

It is well-established that glia plays a pivotal role in the maintenance of brain energy homeostasis and the modulation of neuroinflammation. This fundamental process is critical in the development of numerous neurological diseases [153]. Neuroinflammation, orchestrated by the activation of microglia and astrocytes, signifies the brain’s response to insults such as damage, infection, or metabolic dysfunction. This process is associated with neurodegenerative diseases including Alzheimer’s disease, Parkinson’s disease, or Multiple Sclerosis, among others [154,155].

In this context, glycogen metabolism plays a pivotal role in regulating neuroinflammation. Astrocytes, responsible for storing glycogen, are also activated during inflammatory processes, and this metabolism is altered in states of neuroinflammation [156]. The release of lactate and other metabolites derived from glycogen can modulate the inflammatory response of glia, favoring a protective environment for neurons [157,158]. However, in pathological conditions, such as neurodegeneration, glycogen metabolism in astrocytes can become dysfunctional, resulting in prolonged glia activation and exacerbation of neuroinflammation, which, in turn, aggravates neuronal damage [159].

Abnormal glycogen accumulation in astrocytes has been found to be associated with a chronic inflammatory response that contributes to the progression of diseases such as Alzheimer’s and Parkinson’s [160]. The disruption of glial metabolism affects the energy function and the ability of astrocytes to regulate the brain’s immune response, which can generate a cyclical process of inflammation and neuronal damage [161]. One potential therapeutic strategy to reduce neuroinflammation and protect neuronal health in these conditions is to modulate glycogen metabolism [162].

### 6.2. Recent Hypotheses Concerning Neuroglial Dysfunction in Pathologies

Recent research has begun to explore the role of neuroglial dysfunction as a central factor in the development of various neurological pathologies [163]. It has been proposed that disorders in metabolic communication between neurons and glia, particularly in relation to glycogen metabolism, may serve as an early trigger for neurodegenerative diseases [164,165]. A hypothesis that is currently gaining ground suggests that alterations in glycogen metabolism in astrocytes compromise the energy supply to neurons and generate a metabolically unsustainable environment for glial cells [166,167]. This phenomenon has been shown to promote chronic activation of microglia, thereby contributing to neuroinflammation [168].

Moreover, the failure of neuroglial communication has been posited as a factor contributing to the accumulation of toxic proteins, including amyloid-beta in Alzheimer’s and alpha-synuclein in Parkinson’s, by modifying the functions of glial cells responsible for the clearance of these protein aggregates. Dysfunction in glycogen recycling and lactate production has the potential to interfere with autophagy and protein degradation mechanisms in glial cells, resulting in an accumulation of toxic waste within the brain [169,170].

A different research direction has proposed that the impaired brain energy homeostasis, brought about by neuroglial dysfunction, may be associated with alterations in cerebral blood flow, leading to local hypoxia and exacerbating neuronal damage [171]. Moreover, hypoxia has been demonstrated to influence glycogen metabolism and lactate production, thus engendering a detrimental cycle that facilitates the progression of the disease [172,173]. Alterations in this metabolism, particularly in the context of neuroinflammation and neurodegeneration, have been identified as a potentially crucial factor in the neuroglial dysfunction observed in several pathologies.

## 7. Therapeutic Perspectives in the Management of Glycogen Metabolism

In recent decades, research in this field has explored different therapeutic strategies, including enzyme modulation, dietary interventions, gene therapies, and therapies based on the modulation of the intestinal microbiota [174,175].

### 7.1. Enzymatic Modulation as a Therapeutic Strategy

A promising approach to correcting dysfunctions in brain glycogen metabolism entails the selective modulation of key enzymes involved in its synthesis and degradation, such as glycogen synthase and phosphorylase [176]. These enzymes regulate the ability of astrocytes and other CNS cells to store and mobilize glycogen, which has a direct impact on brain energy homeostasis [177].

In recent years, significant advancements in preclinical research have led to the identification of various selective inhibitors and activators of these enzymes, suggesting potential therapeutic interventions for diseases involving altered glycogen metabolism [178]. For instance, in the field of epilepsy, the investigation of glycogen synthase inhibitors has emerged as a promising approach to enhance brain glycogen levels and prevent episodes of neuronal hypoglycemia, a condition that can contribute to excessive neuronal excitability [179,180]. Similarly, studies have demonstrated that phosphorylase activation can increase glycogen degradation in astrocytes, thereby providing an additional source of lactate to neurons during periods of increased metabolic activity [181,182]. In this regard, recent studies have investigated the potential of glycogen synthase inhibitors in Alzheimer’s disease as a means to restore metabolic balance in the brain and mitigate cognitive deficits associated with impaired glycogen metabolism [183].

It is imperative to acknowledge the persistent challenges in implementing this approach in human models. The specificity and long-term safety of enzyme modulators necessitate meticulous evaluation. A non-selective disruption of glycogen metabolism could potentially induce deleterious effects on specific cellular functions and global brain homeostasis.

### 7.2. Gene Therapies and Innovative Molecules

The advent of gene therapy has given rise to novel approaches aimed at rectifying defects in brain glycogen metabolism at the molecular level. The novel therapeutic interventions encompass the implementation of genes that encode pivotal enzymes in glycogen metabolism, such as glycogen synthase or phosphorylase, into glial or neuronal cells. The objective of this intervention is to reinstate optimal glycogen function within the brain, while concomitantly enhancing its expression and activity in specific neuronal and glial cells [184]. The restoration of metabolic functionality in these cells has the potential to alleviate energy deficits and concurrently counteract neurodegenerative processes [185,186]. Notable gene therapy studies in models of glycogen storage diseases have demonstrated the ability to restore normal glycogen storage and utilization in glial cells, leading to improved cognitive function and overall brain health [187].

Conversely, the emergence of innovative molecules as a complementary or alternative therapeutic modality is receiving increased attention. These molecules possess the capacity to directly regulate glycogen metabolic pathways through diverse mechanisms, including the activation or inhibition of specific enzymatic pathways [188]. A notable example is modulators of AMPK, a protein kinase that plays a central role in the regulation of energy metabolism [189]. The control of this kinase could potentially balance glycogen synthesis and degradation in the brain. Other approaches include the use of compounds designed to interact with epigenetic regulatory elements, such as histones or non-coding RNA, which would allow for more durable and adaptive modulation of genes involved in these metabolic pathways [190,191]. Innovative small molecules targeting enzymes such as GSK-3β, which are involved in glycogen regulation, have also demonstrated efficacy in preclinical models of neurodegenerative diseases [192].

The combination of these therapeutic strategies provides a comprehensive and personalized approach to diseases related to glycogen metabolism. These strategies target not only the correction of underlying genetic abnormalities but also the optimization of the overall metabolic response of brain tissue. These advances promise a significant impact on patient quality of life by providing long-term and potentially curative therapeutic solutions, always considering the need for robust clinical trials to validate their safety and efficacy.

### 7.3. Modulation of the Gut Microbiota—An Emerging Therapeutic Approach in Brain Glycogen Metabolism

Recent scientific findings have provided evidence that the gut microbiota exerts a pivotal regulatory influence on diverse cerebral functions, including those associated with glycogen metabolism [193,194]. This relationship has become increasingly evident in recent years, with research demonstrating how microbiota-derived metabolites can influence brain function, including glial cells responsible for the storage and release of glycogen. Short-chain fatty acids (SCFAs), in particular, have been implicated in the modulation of neuroinflammation, synaptic plasticity, and mitochondrial function, all of which are critical aspects of brain glycogen metabolism [195,196].

Therapies based on the modulation of the intestinal microbiota, such as the use of probiotics, prebiotics, and fecal microbiota transplantation, have been shown to have beneficial effects in a variety of neurological disorders, including epilepsy, Alzheimer’s, and Parkinson’s [197]. These therapeutic interventions aim to restore a healthy balance of microorganisms within the intestinal microbiota, which, in turn, may exert an indirect influence on brain metabolism [198].

Interventions, such as fecal transplantation, have demonstrated a remarkable capacity to restore brain metabolic function in animal models of neurodegenerative diseases. In these models, the restoration of healthy gut microbiota has exerted a favorable influence on the brain’s capacity to regulate its energy metabolism, encompassing glycogen availability and utilization [199,200]. Moreover, preclinical studies suggest that modulation of the microbiota can enhance communication between neurons and astrocytes, thereby facilitating the flow of lactate and other glycogen-derived metabolites to active neurons [195]. Recent studies in Parkinson’s disease models have demonstrated that microbiota modulation through prebiotics can enhance glycogen-derived lactate delivery to neurons, thereby improving motor function and cognitive performance [201].

Despite the evident potential of microbiota-based therapies to treat neurological diseases associated with glycogen metabolism, further research is necessary to fully comprehend the influence of microbiota modulation on brain glycogen-specific processes. The identification of precise molecular mechanisms, the long-term effects of these interventions, and the feasibility of developing personalized treatments are imperative for the successful integration of this approach into clinical practice.

### 7.4. Further Therapeutic Considerations for Glycogen-Related Brain Disorders

**Figure 3:**
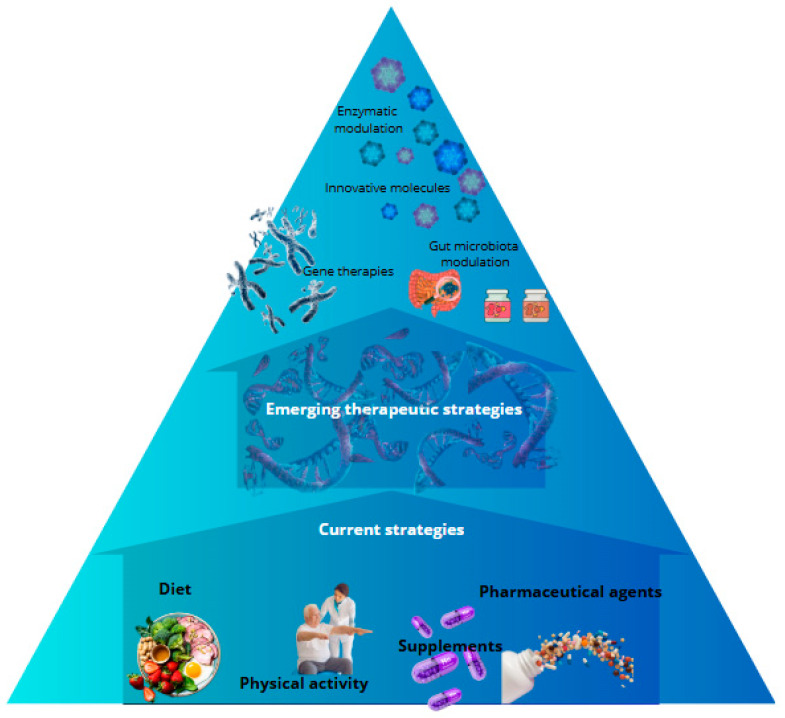
Emerging therapeutic strategies in disorders of brain glycogen metabolism. Principal therapeutic strategies targeting brain glycogen metabolism (gene therapies, enzyme modulation, innovative molecules, and gut microbiota modulation). The objective of these interventions is to correct metabolic defects, restore energy homeostasis, reduce neuroinflammation, and delay neurodegeneration.

In addition to the general therapeutic strategies, there exist other substantial approaches for addressing specific disorders associated with glycogen metabolism in the brain. For instance, in Glycogen Storage Disease Type II (Pompe disease), characterized by glycogen accumulation in lysosomes due to an enzyme deficiency, known as acid alpha-glucosidase, the therapeutic intervention known as enzyme replacement therapy (ERT) holds significant importance. While this therapeutic approach is chiefly focused on addressing muscle dysfunction, recent studies have explored its potential benefits for the central nervous system, particularly in enhancing cognitive functions impaired by glycogen accumulation [202] (Figure 3).

## 8. Conclusions and Future Directions

The study of brain glycogen has advanced significantly in recent decades, revealing its essential role as an energy source and its involvement in more complex functions related to synaptic plasticity, sleep–wake regulation, and neuroglial communication. Currently, numerous gaps in knowledge still persist, especially with regard to its regulation in the human brain and the implications of its alteration in various neurological pathologies. Current information underlines the importance of astrocytes in the storage and mobilization of glycogen, as well as their interaction with neurons under normal and pathological conditions.

One of the key findings of brain glycogen research is that, beyond its role as a rapid energy source, glycogen plays a crucial regulatory role in brain homeostasis. This includes modulation of neuronal activity, response to hypoglycemia, and maintenance of synaptic function, making it an interesting target for therapeutic interventions. Furthermore, the discovery of the relationship between glycogen and the intestinal microbiota has opened new perspectives on how the modulation of the intestinal flora could indirectly influence glycogen metabolism and, therefore, brain health.

Notwithstanding the advances that have been made, there are still significant gaps in the understanding of the specific molecular mechanisms that regulate glycogen metabolism in the brain. The enzymatic regulation of its synthesis and degradation, as well as its interaction with other energy metabolites, remains an area that has been under-explored. Furthermore, current research is often limited to animal models, which raises the need for larger and more specific studies in humans that validate these mechanisms and proposed therapies.

From a methodological perspective, one of the main challenges is the difficulty in studying brain glycogen under physiological and pathological conditions. Although imaging techniques and metabolomic analysis have advanced, current tools remain limited to accurately detecting glycogen concentrations in real time and in specific contexts of the CNS. The development of new technologies that allow more detailed and less invasive visualization of brain glycogen will be crucial to improving our understanding of its function in the brain.

With respect to targeted therapies, interventions grounded in enzyme modulation and metabolic diets demonstrate considerable promise, though they remain to be clinically validated. Gene therapies, which hold the potential to offer a more precise correction of defects in glycogen metabolism, are still in their nascent stages. Their implementation in clinical practice is encumbered by technical and ethical challenges. Similarly, the field of modulating the intestinal microbiota as a therapeutic strategy to regulate brain glycogen metabolism is in its nascent stages and requires further studies, both preclinical and clinical, to establish its possible applications in neurological disorders.

Future research should prioritize the detailed study of the molecular and cellular mechanisms involved in the regulation of glycogen in the brain, as well as the identification of specific biomarkers that can facilitate the diagnosis and monitoring of disorders related to its metabolism. In addition, clinical research should prioritize the evaluation of therapies based on gut microbiota modulation and dietary interventions as complementary approaches in the treatment of neurological diseases. The combination of therapies that address brain energy metabolism comprehensively has the potential to transform the management of various neurological pathologies in the near future.

## Data Availability

No new data were created or analyzed in this study.
